# Determination of Anti-Adeno-Associated Viral Vector Neutralizing Antibodies in Patients With Heart Failure in the Cardiovascular Foundation of Colombia (ANVIAS): Study Protocol

**DOI:** 10.2196/resprot.5535

**Published:** 2016-06-09

**Authors:** Melvin Y Rincon, Carlos E Prada, Marcos Lopez, Victor Castillo, Luis Eduardo Echeverria, Norma Serrano

**Affiliations:** ^1^Centro de InvestigacionesFundacion Cardiovascular de ColombiaFloridablancaColombia; ^2^Department of Gene Therapy and Regenerative MedicineFree University of Brussels (VUB)BrusselsBelgium; ^3^Division of Human GeneticsCincinnati Children’s Hospital Medical Center (CCHMC)Cincinnati, OHUnited States; ^4^Translational Biomedical Research GroupBiotechnology LaboratoriesFundación Cardiovascular de ColombiaFloridablancaColombia; ^5^Graduate Program in Biomedical SciencesFaculty of HealthUniversidad del ValleCaliColombia

**Keywords:** gene therapy, heart failure, adeno-associated viral vector, neutralizing antibodies, Colombia

## Abstract

**Background:**

Recent progress in the pathophysiology of heart failure (HF) has led to the development of new therapeutic options such as gene therapy and the use of adeno-associated viral (AAV) vectors. Despite the promising results in early clinical trials of gene therapy for HF, various obstacles have been faced, such as the presence of neutralizing antibodies (NAbs) against the capsid vectors. NAb activity limits vector transduction levels and therefore diminishes the final therapeutic response. Recent studies evaluating the prevalence of NAbs in various populations found considerable geographic variability for each AAV serotype. However, the levels of NAbs in Latin American populations are unknown, becoming a limiting factor to conducting AAV vector therapeutic trials in this population.

**Objective:**

The goal of this study is to determine for the first time, the prevalence of anti-AAV NAbs for the serotypes 1, 2, and 9 in HF patients from the city of Bucaramanga, Colombia, using the *in vitro* transduction inhibition assay.

**Methods:**

We will conduct a cross-sectional study with patients who periodically attend the HF clinic of the Cardiovascular Foundation of Colombia and healthy volunteers matched for age and sex. For all participants, we will evaluate the NAb levels against serotypes AAV1, AAV2, and AAV9. We will determine NAb levels using the *in vitro* transduction inhibition assay. In addition, participants will answer a survey to evaluate their epidemiological and socioeconomic variables. Participation in the study will be voluntary and all participants will sign an informed consent document before any intervention.

**Results:**

The project is in the first phase: elaboration of case report forms and the informed consent form, and design of the recruitment strategy. Patient recruitment is expected to begin in the spring of 2016. We expect to have preliminary results, including the titer of the viral vectors, multiplicity of infections that we will use for each serotype, and the general validation of the assay, at the end of 2016. The final results are expected mid-2017.

**Conclusions:**

This project is the first effort to evaluate NAb levels against AAV1, AAV2, and AAV9 serotypes in patients with HF in Latin America. Our results will allow us to check the cross-reactivity response between the serotypes assessed, to describe the epidemiological characteristics of the participant population, and to set up a link with earlier reports of NAb prevalence in the literature.

## Introduction

Heart failure (HF) is the cardiovascular disease (CVD) with the highest mortality and morbidity globally. Recent reports indicate that up to 1% to 2% of adult populations in developed countries have HF, with an increasing prevalence of ≥10% among people ≥70 years old [1]. Despite the significant improvement in management of disease symptoms and the decreased disease progression achieved with newly developed pharmacological therapies and invasive procedures, alternative therapeutic approaches are still urgently needed to provide a definitive cure [2]. Recent advances in structural and molecular cardiology have shown that abnormalities in the calcium cycling proteins play a significant role in the induction and progression of HF. Decreased sarcoplasmic reticulum Ca^2+^-ATPase (SERCA2a) activity has been described to be a critical pathway responsible for pathologic modification of the cardiomyocytes during HF [3]. These findings, together with advances in gene transfer technologies, have led to gene therapy being considered as a new therapeutic option for CVDs, and specially for HF. Encouraging results from early clinical trials involving the use of adeno-associated viral (AAV) vectors delivering the *SERCA2* gene have generated high expectations [4]. Additionally, other proteins have shown promising results in preclinical models of HF, such as S100A1 and adenylyl cyclase 6 [5,6].

### Emergence of Gene Therapy as a Therapeutic Option for HF and CVD

Three elements must be considered in the design of a gene therapy strategy: the therapeutic gene that will be cloned into the transgene expression cassette; the target cells or tissues in which the vector will deliver the genetic material; and the viral vector used to facilitate entry of the gene into the target tissue [7]. Viral vectors consist of genetic material surrounded by a protein capsid, which facilitates the transcellular transport and internalization of the therapeutic gene into the target cell [7]. Additionally, the capsid protects the transgene expression cassette from lysosomal degradation during its trafficking to the nucleus [8]. The discovery of viral vectors with cardiomyocyte and endothelial tropism accelerated the progression of gene therapy as a therapeutic option for HF [9]. Novel transductional modifications of the first-generation vectors and translational strategies in the transgene expression cassettes have been developed to achieve higher and longer-term expression of the transfected gene while trying to decrease the amount of adverse effects [10]. As Mingozzi and High state, “The gene is the active agent of therapeutic, but the vector, in most cases derived from a virus, is also a critical determinant of therapeutic success and of the toxicity profile” [11]. Importantly, depending on the viral vector selected, the immune response to the vector or to the cells expressing the modified gene varies and can become a limiting factor for successful therapy [12]. Specific immune responses can prevent vector gene transfer after readministration of the vector, limit the duration of gene expression, or produce an immune response against the genetically modified cells [13].

Viral vectors derived from adenovirus, retrovirus, lentivirus, or AAV have been used as therapeutic tools for a broad spectrum of genetic and nongenetic diseases, including CVD and HF. Lentivirus vectors originate from human immunodeficiency virus 1 and have been used successfully to treat hematopoietic monogenic diseases thanks to their therapeutic long-term effects [14,15]. However, their use in gene therapy applications for CVD or specifically for HF is more limited, given their relatively poor transduction after systemic administration and the risk of insertional mutagenesis [16]. Adenoviral vectors are non-enveloped double-stranded DNA vectors, which are not able to insert the genome into the host cell DNA. Adenovirus serotype 5 has been used predominantly in preclinical and clinical trials of gene therapy for myocardial infarction and ischemic diseases, in which short-term transgenic expression is required [17].

The most commonly used viral vectors for HF and the focus of our project are the AAV vectors. AAV vectors are single-stranded DNA vectors with a favorable safety profile and the ability to achieve long-term transgene expression in a wide range of tissues, including heart [18]. The storage capacity of the AAV vector (up to 4.7 kB) restricts the size of the transgene expression cassette that can be used and needs to be considered beforehand [19]. More than 100 AAV serotypes existing in nature have been reported, many of them with variable tissue tropism, which is determined by the structure of the proteins in the capsid [18]. AAV1, AAV6, AAV8, and AAV9 have been reported as the serotypes with favorable tropism for cardiomyocytes after systemic administration. However, tropism for other cell types, such as hepatocytes, is still present and needs to be carefully considered before any clinical trial [9,20,21]. Satisfactory preclinical results in large experimental animal models with a heart anatomy similar to that of humans led to the clinical translation of AAV-based technology for transgene expression of key proteins involved in the progression of HF, such as SERCA2a [22]. The first clinical trial for HF was conducted using an AAV1 vector, the Calcium Upregulation by Percutaneous Administration of Gene Therapy in Cardiac Disease (CUPID) trial [23]. Simultaneously, two other clinical trials are in the late phases of recruitment, the phase 2 study Investigation of Safety and Feasibility of AAV1/SERCA2a Gene Transfer in Patients With Chronic Heart Failure (SERCA-LVAD; NCT00534703) and the AAV1-CMV-SERCA2a Gene Therapy Trial in Heart Failure (AGENT-HF; NCT01966887), with results expected in 2016.

### Immune Response Against AAV Vectors

Immune responses of T cells against antigens of the AAV capsid presented in association with major histocompatibility complex class I on the surface of transduced target cells can reduce long-term gene expression, after an immune rejection of the genetically modified target cells [11]. Transient immunosuppressive therapies or AAV capsid variants that reduced the presentation of peptides derived from the AAV capsid can decrease this risk of immune responses [24,25]. The vector capsid is, in most cases, an exact replica of the viral capsid, and thus the immune response against the vector may be influenced by previous exposure of the immune system to environmental viruses from which the vector is designed [26]. Humans, early in life, are naturally exposed to wild-type AAV, and consequently the frequency of seropositivity of anti-AAV steadily increases after 2 years of age [26]. Humoral immunity to AAV can also be found in infants, as a result of vertical transmission of anti-AAV antibodies from the mother. In adults, anti-AAV2 antibodies are the most prevalent (up to 70% of healthy humans), followed by serotypes such as AAV5, AAV9, and AAV8, which are much less frequent [27]. Although the risk of inflammatory immune responses after AAV vector transduction is significantly lower than with adenoviral vectors, there are still significant immunological complications that must be evaluated. In particular, the high prevalence of preexisting anti-AAV neutralizing antibodies (NAbs) in the population may result in rapid neutralization of vectors derived from AAV, preventing adequate gene transfer [12]. Similarly, the induction of NAbs after AAV-based gene therapy impedes adequate therapeutic response following readministration of AAV vectors [7,28].

### Anti-AAV NAbs

There is a high degree of amino acid sequence conservation between AAV capsids, leading to anti-AAV antibody cross-reactivity in a wide range of serotypes [29]. Although antibodies to AAV2 are clearly the most prevalent in humans (up to 70%), the natural host for this serotype, antibodies that recognize virtually all serotypes of AAV may be found in a high proportion of humans [26]. Studies have shown that, after AAV vectors were introduced into the systemic circulation in humans, the preexisting humoral immunity to AAV vectors profoundly reduced transduction efficiency in the target tissue. Results of the first clinical trial for hemophilia B showed that relatively low titers of NAbs, as low as 1:17, can completely neutralize large vector doses, without detection of transgene expression [12]. The existence of anti-AAV NAbs is one of the main limitations faced in gene therapy clinical trials after intravascular delivery of AAV vectors. In the CUPID 2 trial, patients with high levels of NAbs were excluded. Of the 509 patients initially evaluated, >50% were excluded for presenting levels of NAbs outside of the safety range established in the study [30], suggesting the importance of prior determination of anti-AAV NAb circulating levels in different populations.

The prevalence of NAbs against AAV in humans was initially reported in the 1960s and 1970s, mainly anti-AAV1 and -AAV2 serotypes, the only known serotypes at that time. NAb variation is estimated at between 30% and 80% based on previously published studies in other populations [31,32]. More recent studies have evaluated the prevalence of NAbs in the most commonly used serotypes for preclinical studies (AAV5, AAV7, AAV8, and AAV9) and compared them with previously described serotypes. One study that collected samples from 10 countries on 4 continents (United States, Europe, Africa, and Australia) showed a prevalence of NAbs to AAV2 of between 30% and 60% of the total population [27]. This was significantly higher than the prevalences of NAbs to AAV7, AAV8, and AAV9 serotypes, which were reported to be between 15% and 30%. Notably, higher frequencies of NAbs were observed for all AAV serotypes in Africa than in the other regions [27]. However, none of the existing reported studies have included a Latin American population.

An interesting finding is the significant variability in the prevalence of anti-AAV NAbs, influenced by the geographic origin of the population. While the prevalence of NAbs against AAV1 in Africa and China is around 50% to 70%, in other countries such as Belgium, Greece, Italy, and the United States, it is only 20% to 30% [27]. Overall, the prevalence of anti-AAV NAbs appears to be greater in developing countries. Factors such as living conditions, population density, hygienic conditions, different levels of health care, and the AAV detection method for NAbs can influence these results; in particular, the prevalence of anti-AAV1 NAbs was higher in women than in men [27]. The health status of the target population can also affect the prevalence NAbs against AAV, especially in those patients with a compromised immune system. These people had a lower prevalence of anti-AAV NAbs than that in the healthy population [26,27,33,34]; furthermore, the prevalence of NAbs was higher in elderly than in younger populations [35].

### AAV Antibody Determination

Diverse methods have been developed to detect antibodies against different serotypes of AAV. Some of them are based on total binding antibodies to the AAV capsid, and others on the functional determination of antibodies that neutralize AAV vector transduction *in vitro* or *in vivo* [29]. The first reports, in the 1970s, were based on binding of antibodies to AAV capsid vector evaluated by enzyme-linked immunosorbent assay (ELISA) and Western blot [32]. These studies focused on AAV1 and AAV2. The discovery in the last decade of new AAV vectors required more accurate assays to evaluate not only the level of binding but also the effect of the specific NAb for each AAV serotype on vector activity. Given the profound effect of anti-AAV antibody on transduction efficiency, the development of sensitive and reliable methods for measuring patients’ titers of NAbs is critical before starting a clinical trial with gene therapy. Furthermore, the method of choice must ensure correlation of titers with the clinical outcome reported for gene therapy.

#### Elisa

ELISA is based on a capture assay in which the entire AAV capsid or peptides coated on a plate and AAV immunoglobulin antibodies present in serum are detected with a secondary antibody. ELISAs are easier to configure and are relatively sensitive at determining antibody levels. However, the total amount of anti-AAV antibodies is not always proportional to its neutralizing activity, especially in people with low titers, making this assay unreliable for determining the eligibility of people for their participation in clinical trials with AAV vectors.

#### In Vitro Cell-Based Assays

These are among the most widely used methods to determine anti-AAV NAbs [27,36,37]. Specifically, the *in vitro* transduction inhibition assay has become the standard test to evaluate the presence of anti-AAV NAbs [37]. Typically, an AAV vector expressing a reporter gene is mixed with serially diluted amounts of the test sample, and this vector serum mixture is incubated with the cell line of choice, which is then analyzed for reporter gene expression. The starting dilution of the test sample, which defines assay sensitivity, varies across studies, ranging from 1:2 to 1:20. The NAb50 titer is reported as the highest serum dilution that inhibits the transduction of the reporter gene by 50% [37]. While NAb *in vitro* assays are relatively easy to set up and give consistent results, they have some limitations: the most important derives from the fact that most of the AAV serotypes are highly inefficient for *in vitro* cell transduction. This limitation can be at least partially overcome with the use of reporter genes with high detection sensitivity (eg, luciferase) and cell lines more permissive for AAV transduction [37]. In addition, several related parameters of the cell culture conditions can contribute to variability of the assay, such as the cell line used, cell density, and the number of passages of cells during cultivation.

In summary, recent reports highlight the importance of identifying the prevalence of NAbs for each AAV serotype in various human populations, due to the serotypes’ geographic variability and their major role in selection of participants for AAV vector clinical trials. This increases the importance of conducting NAb prevalence studies in the Latin American population. Considering the major impact of CVD on the health care systems of developing countries, we intend to determine the prevalence of anti-AAV vector NAbs for the serotypes AAV1, AAV2, and AAV9 in patients with HF in the Cardiovascular Foundation of Colombia (FCV) by the *in vitro* transduction inhibition assay.

## Methods

We will conduct a cross-sectional study to evaluate NAbs against AAV1, AAV2, and AAV9 serotypes in patients with HF at the FCV-HF clinic. Patients who fulfill the inclusion criteria will be invited to participate in the study. Those who accept will be asked to read and ask the questions they deem relevant to informed consent before signing the form (see Multimedia Appendix 1). Once the patients consent to participate, a standardized and approved survey to evaluate epidemiologic and socioeconomic variables and major CVD risk factors will be applied and complemented with relevant clinical information obtained from the electronic medical record system (SAHI 2.0 software, integrated hospital management system) developed by the FCV. A physical examination will be performed, including evaluation of blood pressure levels, anthropometric measurements, and a record of the last echocardiographic parameters available. Finally, a sample of peripheral blood will be collected from each participant to determine the presence of anti-AAV NAbs.

### Study Population

We will evaluate 60 patients with a diagnosis of HF (American Heart Association stage B or higher criteria) followed in the FCV-HF clinic (see Textbox 1 for inclusion criteria for the patient group). All adult patients (≥18 years old) followed in the HF clinic at FCV will be eligible for the study. We will use various methods to recruit patients, including electronic clinical data from the SAHI 2.0 records. Additionally, we will invite 60 healthy volunteers, matched by age and sex, to participate. Participants will be considered as healthy volunteers if they are ≥18 years old, nationals from a Latin America country with a residence address in Colombia, and not meeting any of the exclusion criteria detailed in Textbox 2. We selected the number of participants as convenience size due to the internal financial and technical limitations of the project.

**Inclusion criteria for patients in the ANVIAS study.**

- Age ≥18 years
- Diagnosis of heart failure stage B or higher according to the American Heart Association criteria
- National of a Latin America country with residence address in Colombia
- Followed in the Cardiovascular Foundation of Colombia heart failure clinic

**Exclusion criteria for patients simultaneously presenting with heart failure and for volunteer participants the ANVIAS study.**

- Multiple blood transfusions
- Recent plasmapheresis
- Infection with human immunodeficiency virus
- Immunosuppression or recent treatment with corticosteroids
- Solid organ or bone marrow transplantation
- Diagnosis of Chagas disease
- Pregnancy
- Preexisting condition of asthma, obstructive sleep apnea, or chronic obstructive pulmonary disease
- Prior hormone replacement therapy
- Heavy drinking, defined as the consumption of ≥5 alcoholic drinks on the same occasion on each of ≥5 days in the past 30 days (US Substance Abuse and Mental Health Services Administration definition)

### Storage of Biological Material

Participants will be asked to consent to donate biological material to be stored according to all quality and ethical requirements in the biobank of the FCV. The FCV biobank is a biomedical platform for future research, which is of great relevance in contributing to establishing bridges between basic, translational, and clinical research, and health care practice. Importantly, participants in this proposed study will be required to grant additional consent for taking and storing samples in the biobank (see Multimedia Appendix 1).

### Blood Sample Collection and Separation of Components

Samples of peripheral blood will be collected by antecubital venipuncture with a Vacutainer system (Becton, Dickinson and Company, Franklin Lakes, NJ, USA) into 2 tubes containing 4 mL of EDTA anticoagulant and labelled to identify the participant. Then, the sample will be centrifuged to separate the components and stored according to established protocols by the FCV biobank. A physical and digital date and time record of sampling separation and storage components will be kept, as well as the name of the person responsible for the procedures.

Samples will be handled from the first to the final phase by the following FCV biobank storage steps: 1) obtaining informed consent for storage of samples in the biobank: Protocol PR-BIO-01, 2) blood sampling: Protocol PR-BIO-03, 3) transporting biological samples: Protocol PR-BIO-02, 4) receiving and storing samples: Protocol PR-BIO-05, 5) quality control of human biological material, storage, and registration: Protocol PR-BIO-08.

### Data Processing and Quality Control

The data will be recorded on case report forms previously designed for that purpose. After completing the case report form, the monitor of the study will review the data to confirm completeness, readability, accuracy, and consistency. All detected errors will be reported, evaluated, and properly corrected by the originator of the forms. All corrections will be noted with dates and initials of the person issuing the correction in accordance with the World Health Organization’s Guidelines for Good Clinical Practice [38]. Immediately after the form is correct, the information will be recorded in a database.

### Production and Purification of Viral Vectors

We will produce the AAV1, AAV2, and AAV9 vectors using the cotransfection strategy of human embryonic kidney 293 cells specially modified to facilitate transfection of AAV (AAV-293; Agilent Technologies, Santa Clara, CA, USA; catalog #240073) via calcium phosphate (ThermoFisher Scientific, Waltham, MA, USA) [39]. We will use a pAAV with the plasmid of interest, a helper adenoviral plasmid, and a chimeric construct having the AAV2 *rep* gene, along with the corresponding AAV1, AAV2, or AAV9 *cap* gene [9].

We will evaluate the AAV-293 cells for microbial contamination using the polymerase chain reaction protocol for detecting mycoplasma. Following the previously described protocol, 2 days after transfection, we will collect cells and purify the vector particles using precipitation gradient centrifugation methods. The harvested cells will be lysed by successive cycles of freezing, thawing, and sonication, then treated with Benzonase nuclease (Novagen, Madison, WI, USA) and deoxycholic acid (Sigma-Aldrich, St. Louis, MO, USA), and then subjected to 3 successive rounds of cesium chloride density-gradient ultracentrifugation (ThermoFisher Scientific). We will collect the fractions containing AAV vector, concentrate it using 1-mmol/L MgCl_2_(phosphate buffered saline) (Gibco, BRL), and store it at –80°C until use. Quantification of AAV1, AAV2, or AAV9-cytomegalovirus-luciferase (in viral genomes/mL) vectors will be determined by quantitative real-time polymerase chain reaction probes using SYBR Green Supermix (Bio-Rad, Hercules, CA, USA) and primers specific for luciferase complementary DNA. The forward and reverse primers that we will use are CCCACCGTCGTATTCGTGAG5'-3' and 5'TCAGGGCGATGGTTTTGTCCC-3', respectively.

Typically, we have managed to standardize the protocol and achieve titers in the range of 2×10^12^ to 5×10^12^ viral genomes/mL. To generate standard curves, we will use a plasmid of known copy numbers corresponding to the vectors used to generate AAV vectors carrying the appropriate complementary DNA. Viral vectors will be produced in collaboration with the laboratory of the Department of Gene Therapy and Regenerative Medicine, Free University of Brussels, by the principal investigator, Dr Melvin Rincon.

### Determination of Neutralizing Antibodies

We will determine NAb titers against serotypes AAV1, AAV2, and AAV9 following the recently published protocol in *Human Gene Therapy Methods* by Dr F Mingozzi’s group [37]. This protocol is used due to high reproducibility of the results, in addition to the efficiency and safety it offers. Determination of NAb titers is based on an AAV vector expressing a reporter gene, which is incubated with the sample to be evaluated before *in vitro* transduction of a specific cell line. We describe the main features of the protocol below; for a more detailed protocol please review the publication by Meliani et al [37].

### Multiplicity of Infection Selection

Importantly, as Meliani et al [37] mention, an optimal multiplicity of infection (MOI) should be determined for each serotype. We will select the MOI for each serotype NAb assay by determining the viral vector needed to give a luciferase signal above background signal and below saturation levels, measured in relative light units, considering that the number of AAV genome copy particles to be used is equal to the desired MOI multiplied by the number of cells to be infected.

For our project, we will transduce human embryonic kidney 293-derived 2V6.11 cells with an increasing MOI of the corresponding serotype (AAV1, AAV2, or AAV9); 24 hours later we will measure luciferase expression and select the most appropriate MOI for each serotype.

### Assay Validation

Validation of any assay is fundamental before translation to clinical practice. Simultaneously with execution of the project and, based on initial results from the patients’ samples, we will perform a process similar to the following (to be reported in the final paper): 1) determine the optimal cut point (threshold), 2) determine sensitivity, 3) select quality controls, 4) set specificity and assay precision, and 5) determine selectivity. We refer the reader to previously published papers for more detailed information [40,41].

After selecting the desired MOI and establishing the conditions of the assay, we will conduct a 4-day NAb assay protocol, as follows.

#### Day 1: Cell Culture 2V6.11

We will suspend 2×10^4^ 2V6.11 cells in 100 μL of Dulbecco’s modified Eagle’s medium (DMEM)/Ham’s nutrient mixture F-12 culture medium (Sigma-Aldrich) supplemented with 10% fetal bovine serum. The cells will be cultured for 24 hours in an incubator (37°C, 5% CO_2_) in 96-well culture plates. To induce expression of the *E4* gene, Ponasterone A will be added to the culture medium (Sigma-Aldrich) (10 mL).

#### Day 2: Sample Controls and AAV Vector Preparation

For transduction, we will prepare serial dilutions of 10 mL of patient serum and a control sample using fetal bovine serum as the diluent (Table 1). One control sample will correspond to 100% transduction of the vector and another to 0% transduction of the vector to allow for determining the background luminescence signal. The viral vector will be diluted to the concentration selected (according to the desired working MOI) in DMEM without the use of fetal bovine serum. Subsequently, we will add 20 μL of the vector to each of the sample dilutions and controls. These samples will be grown for 1 hour at 37°C and, subsequently, 7.5 μL of each neutralized sample will be added to the cell culture medium. Cells will remain in overnight incubation at 37°C and 5% CO_2_.

**Table 1:** Example^a^ of half-log dilution preparation for the test sample and control using fetal bovine serum as the diluent before being mixed with the viral vector (20 μL) and added to 96-well plated cultured cells.

Dilution no.	Relation	Volume (μL) of sample/control	Volume (μL) of diluent
1	1:1	40	0
2	1:3.16	12 of dilution 1	26
3	1:10	12 of dilution 2	26	
4	1:31.6	12 of dilution 3	26
5	1:100	12 of dilution 4	26
6	1:316	12 of dilution 5	26
7	1:1000	12 of dilution 6	26
8	1:3160	12 of dilution 7	26

^a^Modified from Meliani et al [37].

#### Day 3: Cell Lysis and Measurement of Luciferase Activity

On this day, we will use the protocol for the Bright-Glo Luciferase Assay System (Promega Corporation, Madison, WI, USA). After incubating the assay for 15 minutes at room temperature, we will add 100 μL of cell lysis medium and the luminescence substrate, then wait for 3 minutes until lysis is complete. Luminescence will be measured using the EnSpire reader (PerkinElmer, Inc, Waltham, MA, USA).

#### Day 4: Determination of Anti-AAV NAb titers

To calculate the luciferase expression percentage (LEP) and luciferase inhibition percentage (LIP), we will use the formulas LEP=[(test sample luciferase reading−no virus luciferase signal)/(maximum luciferase signal−no virus luciferase signal)]×100 and LIP = 100 – LEP.

The neutralizing titer of the sample is reported as the first dilution at which ≥50% inhibition of luciferase expression is measured. For example, if ≥50% inhibition is observed at a sample dilution of 1:10, then the neutralizing titer is reported as 1:10. NAb titers will be determined for the selected serotypes in collaboration with the laboratory of the Department of Gene Therapy and Regenerative Medicine at the Free University of Brussels by the principal investigator, Dr Melvin Rincon.

### Statistical Analysis

We will describe data from all measurements of continuous variables using measures of central tendency such as means and standard deviations, considering the coefficients of variation between replicates (3) of the experiments. We will report counts and proportions of categorical variables with their respective 95% confidence intervals. For tests between study groups, we will use analysis of variance to assess differences between means and the chi-square test for differences between proportions. Differences with a value of *P*<.05 will be considered significant. We will use Stata 8.0 statistical software (StataCorp LP).

## Results

We have completed the first phase of the project. During this stage, all the documents necessary for recording and storing information have been elaborated and submitted to the ethical committee of the FCV for approval. We expect to begin recruiting patients in the spring of 2016. We expect to complete the second phase, in which we will validate all the protocols for the project—including determining the titer of the viral vectors, selecting the MOI that we will use for each serotype, and validating the assay—by the end of 2016. Finally, we will conduct the third phase, in which we will analyze serum samples from patients and healthy volunteers, during 2017. We expect to have the first results in mid-2017.

## Discussion

For this project, we will evaluate anti-AAV1, -AAV2, and -AAV9 NAbs by using the *in vitro* transduction inhibition assay, to determine the presence, activity, and titers of NAbs in patient sera. Several critical steps and validation of the protocol should precede analysis of the patient samples (eg, selection of MOI, selection of the cells to be used during the assay). We expect that the final results may indicate the seroprevalence of NAbs for the selected serotypes and allow for comparison with previously reported results from other populations. The causes of the demonstrated high variability between the evaluated populations continue to be debated, and more studies including different healthy populations and under specific pathologic circumstances need to be conducted worldwide. Importantly, the method of determination should be standardized to obtain more reliable and comparable data. Recent reports support the higher sensitivity of intramuscular *in vivo* assays to determine NAb response [42], suggesting that *in vivo* NAb assays are more sensitive than *in vitro* assays for determining inclusion and exclusion criteria in clinical trials; however, the technical difficulties and higher cost associated with using *in vivo* assays in population studies mean that *in vitro* assays still have an important function.

### Conclusion

We expect to have preliminary results, including the titer of the viral vectors, the MOI that we will use, and test assay validations, at the end of 2016. The results of this study will become the initial step for bigger population studies to characterize our population and provide the necessary epidemiological information for clinical trials using AAV in Latin America.

## Abbreviations

AAV: adeno-associated virus
CUPID: Calcium Upregulation by Percutaneous Administration of Gene Therapy in Cardiac Disease
CVD: cardiovascular disease
ELISA: enzyme-linked immunosorbent assay
FCV: Cardiovascular Foundation of Colombia
HF: heart failure
LEP: luciferase expression percentage
LIP: luciferase inhibition percentage
MOI: multiplicity of infection
NAb: neutralizing antibody
SERCA2a: sarcoplasmic reticulum Ca2+-ATPase
