# Supplementary material for: Determination of Anti-Adeno-Associated Viral Vector Neutralizing Antibodies in Patients With Heart Failure in the Cardiovascular Foundation of Colombia (ANVIAS): Study Protocol
Source: JMIR Res Protoc. 2016 Jun 9;5(2):e102. doi: 10.2196/resprot.5535 (PMC4919550; doi:10.2196/resprot.5535)
Supplement: Supplementary file 1 [file resprot_v5i2e102_app1.pdf]

Paciente Nº: ________________________

Consentimiento Informado.
Proyecto: DETERMINACION DE ANTICUERPOS NEUTRALIZANTES CONTRA VECTORES VIRALES ADENO-ASOCIADOS EN PACIENTES CON FALLA CARDIACA EN LA FUNDACION CARDIOVASCULAR DE COLOMBIA (ANVIAS).

INTRODUCCIÓN

La Fundación Cardiovascular de Colombia (FCV), en asocio con Departamento de Terapia Génica y Medicina Regenerativa – Universidad Libre de Bruselas están adelantando un estudio para evaluar los niveles de *anticuerpos neutralizantes (NAb)*, que son células de defensa que se producen naturalmente en nuestro organismo cuando nos exponemos ante una infección (virus, bacteria, hongos). Estos anticuerpos que vamos a medir, los Nab, se produce sólo si, en alguna etapa de nuestra vida estuvimos expuestos a una clase de virus que se denominan virus adeno-asociados los cuales son muy frecuentes en el ambiente.

¿*Por qué medir estos anticuerpos*? Es importante adelantar estos estudios, dado que día a día crece el número de pacientes con *Falla Cardiaca*, que es la incapacidad que tiene el corazón de mandar sangre de manera adecuada a todos los órganos, produciendo incapacidad para quienes la sufren para las actividades de la vida cotidiana, por ejemplo, dificultad para caminar grandes distancias, subir escaleras, hacer ejercicio entre otras.

Los pacientes con falla cardiaca usualmente terminan en trasplante de corazón, por tanto, actualmente se están desarrollando nuevas forma de tratamiento, dentro de la cuales se encuentra la *Terapia Génica*. Este tipo de tratamiento consiste en poner en su organismo genes nuevos capaces de reparar los órganos dañados, para este caso el corazón. Estos nuevos genes viajan hasta el tejido dañado en un vehículo que se llama vector, y que es construido en el laboratorio a partir de un virus. Por lo tanto las personas que tienen estos anticuerpos (Nab), no serían candidatos a recibir este tipo de terapia, pues los anticuerpos atacarían al vehículo o vector antes de llegar a hacer su efecto benéfico en el órgano afectado, por ejemplo, el corazón para los pacientes con falla cardiaca.

No conocemos si los colombianos, y especial los que sufren falla cardiaca, sean positivos o negativos para los anticuerpos contra este virus, y por lo tanto si son candidatos futuros a beneficiarse con este tipo de terapia genética, que se proyecta como una excelente opción de tratamiento para los paciente que sufren de falla cardiaca.

BENEFICIOS

Es posible que los resultados del presente estudio no lo beneficie a usted de manera inmediata, pues aún se requieren años de investigación en Terapia Génica en Falla Cardiaca. Pero en un futuro mediato, el conocimiento que se genera por este estudio nos permitirá seleccionar los pacientes que son candidatos a recibir este tipo de tratamiento.

OBJETIVO DEL ESTUDIO

Determinar la prevalencia de anticuerpos neutralizantes contra los vectores virales AAV serotipos 1, 2 y 9 en una muestra de la población con falla cardiaca de la ciudad de Bucaramanga usando el ensayo de inhibición de la transducción *in vitro* y comparar con la población sana de la ciudad de Bucaramanga

PARTICIPACIÓN

Es importante para nosotros que usted comprenda que con su participación en este estudio, no recibirá ningún tipo de tratamiento, ni prueba diagnóstica. Solo le mediremos sí en su organismos existe o no células de defensa denominadas *anticuerpos Nab.*

Si usted decide acompañarnos en este estudio, un profesional de salud (enfermera) le tomará algunos datos relaciones con su edad, ocupación, vivienda y accederá a su historia clínica para tomar datos relacionados con su condición de salud. Posteriormente le tomará una muestra de sangre, no mayor a

mediciones por su complejidad se harán en el Laboratorio del Departamento de Terapia Génica y Medicina Regenerativa – Universidad Libre de Bruselas – Bélgica, por lo tanto parte de la muestra debe salir del país (Colombia), y una parte se quedará en nuestro laboratorio para en un futuro confirmar los resultados obtenidos en Bélgica y sí usted lo autoriza, para hacer estudios futuros relacionados con este tema.

GARANTÍAS PARA SU PARTICIPACIÓN

Todos sus datos de identificación, resultados y análisis del estudio serán mantenidos en anonimato. Esto significa que su identidad (nombre) NUNCA será dada a conocer.

La decisión de participar es totalmente voluntaria, sin costo alguno. Sí usted decide participar en el estudio, tiene la libertad de retirarse cuando lo desee, y de igual forma de ordenar que su muestra de sangre sea descartada. La no participación en el estudio o el retiro del mismo NO conllevarán a perjuicios de tipo laboral, social, económico o en la relación que usted mantiene con su médico. La información generada en esta investigación es estrictamente confidencial y a ella sólo tendrán acceso los investigadores. Los resultados generales del estudio se publicarán y serán presentados en congresos, pero en ningún caso usted serán identificado personalmente.

Es importante aclarar que ninguna persona involucrada en el estudio, incluyéndolo a usted, recibirá beneficio económico alguno, como pago por su participación.

Nosotros le entregaremos fiel copia de este documento y si tiene alguna duda sobre el mismo, por favor no dude en comentarla con nosotros, quienes estaremos atentos a aclarará sus inquietudes.

CONTACTO

Si usted tiene alguna duda sobre este estudio o necesita información adicional, puede contactar a los doctores, Melvin Rincón al número celular 315-4467410 o a Norma C. Serrano al número celular 310-2740099. Sí por alguna razón no responden de manera inmediata le pedimos el favor de dejar un mensaje de voz o enviar un mensaje de texto, que las investigadores se pondrán en contacto con usted en el menor tiempo posible.

CONSENTIMIENTO

Con mi firma declaro que los alcances del estudio me han sido explicados, que todas mis preguntas fueron respondidas y que los posibles daños o molestias o posibles beneficios me han sido explicados. Entiendo que tengo el derecho de no participar o de dejar de participar en cualquier momento, sin que por ello sufra alguna consecuencia en la atención médica, y que la información que pueda identificarme no será publicada ni presentada en congresos. Yo, por lo tanto, doy mi autorización para participar junto con mi hijo en el estudio: ***DETERMINACION DE ANTICUERPOS NEUTRALIZANTES CONTRA VECTORES VIRALES ADENO-ASOCIADOS EN PACIENTES CON FALLA CARDIACA EN LA FUNDACION CARDIOVASCULAR DE COLOMBIA (ANVIAS).***

Nombre de la participante:____

CC No. ____________________ de _________________________Fecha: día/______Mes/_____Año/_________

Firma____

Nombre del Testigo________________________ CC No. _____________ de _________________________

Firma del testigo _______________________ Fecha día/_____/Mes/_______/Año/________

Yo __________________________ con CC: ______________________________de manera voluntaria autorizo almacenar mi muestra de sangre en el Biobanco de la Fundación Cardiovascular de Colombia FCV para ser utilizados en estudio futuros relacionados solo con investigar anticuerpos relacionados con

Sí la respuesta es NO, la muestra será almacenada por un máximo de 10 años, cumplidos 10 años será desechada la muestra total o lo que quede de ella.

2. Utilizada para estudios secundarios que investiguen anticuerpos relacionados con Terapia Génica, y que los estudios sean realizados solo en Colombia SI_____ NO_____

3. Utilizada para estudios secundarios que investiguen anticuerpos relacionados con Terapia Génica y que los estudios sean realizados con colaboración de países diferentes a Colombia, por lo tanto que la muestra sea sacada del país para análisis especiales SI_____ NO_____

Firma de participante ______________________ CC ________________ Fecha; día/___/Mes/____/Año/____

Nombre del Testigo________________________ CC No. _____________ de _________________________

Firma del testigo _______________________ Fecha día/_____/Mes/_______/Año/_________

Nombre de la persona que obtuvo el consentimiento: ____

Firma _________________________CC No.___________________ Fecha: día/____Mes/_____/Año/_________

PREGUNTAS REALIZADAS POR LOS PARTICIPANTES:
____
____
____
____

Nota: El presente consentimiento informado fue evaluado y aprobado por el Comité de Ética en Investigaciones (CEI), de la Fundación Cardiovascular de Colombia, el cual tiene como domicilio Fundación Cardiovascular de Colombia Calle 155A # 23-58 Urb. El Bosque, Sector E1 Floridablanca – Santander Tel: (07) 6396767 – 6399292
